# Harnessing Surface-Enhanced Raman Spectroscopy for Breath-Based Diagnostics

**DOI:** 10.1021/acs.analchem.5c00167

**Published:** 2025-05-09

**Authors:** Ivan A. Lujan-Cabrera, Eden Morales-Narváez

**Affiliations:** Biophotonic Nanosensors Laboratory, Centro de Física Aplicada y Tecnología Avanzada (CFATA), 73403 Universidad Nacional Autónoma de México (UNAM), Querétaro 76230, Mexico

## Abstract

Human breath contains a myriad of analytes that are associated with human health conditions, making it a noninvasive source of biomarkers. Researchers and engineers are pushing the boundaries of breath analysis for the next generation of diagnostics. Breath analysis with surface enhanced Raman spectroscopy, which allows for the identification of molecular fingerprints in a simple way, represents a powerful platform for the next generation of sensors which are expected to provide tools for personalized healthcare and preventive medicine.

## Graphical abstract

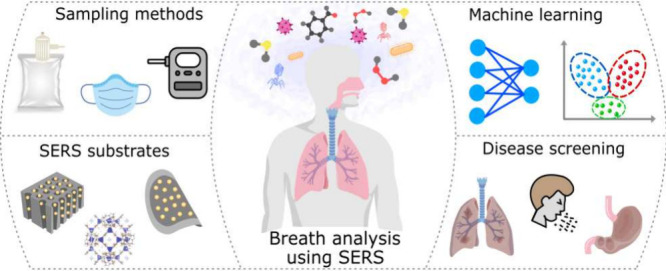

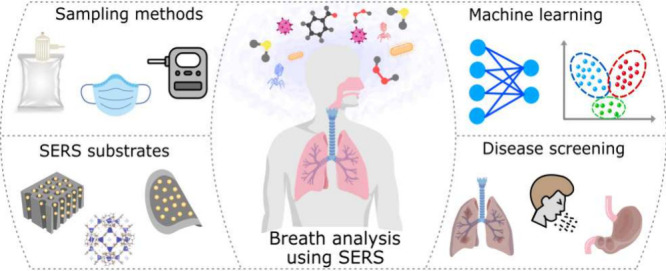

## Introduction

Breath analysis (BA) has recently emerged as a promising alternative for point-of-care diagnostic tools due to its cost-effectiveness, real-time results, noninvasive nature, and ease of sample collection.^1^ BA is an ancient diagnostic technique that took off in the 1780s when over 200 volatile organic compounds (VOCs) were identified in exhaled human breath.^2^ This was an important breakthrough because it was demonstrated that the breath was a complex mixture of different compounds, with more than 3000 VOCs having recently been identified.^3^ Additionally, it was shown that the breath can be separated into two phases, gas and condensate, each providing different types of compounds and analytes.^4^ In the gaseous phase, VOCs can be found and have been used to diagnose different types of cancer. Breath aerosols contain proteins, viruses, and bacteria. Hence, breath represents a source of biomarkers to diagnose various physiological and pathological conditions at different stages, see Figure 1.

**Figure 1:**
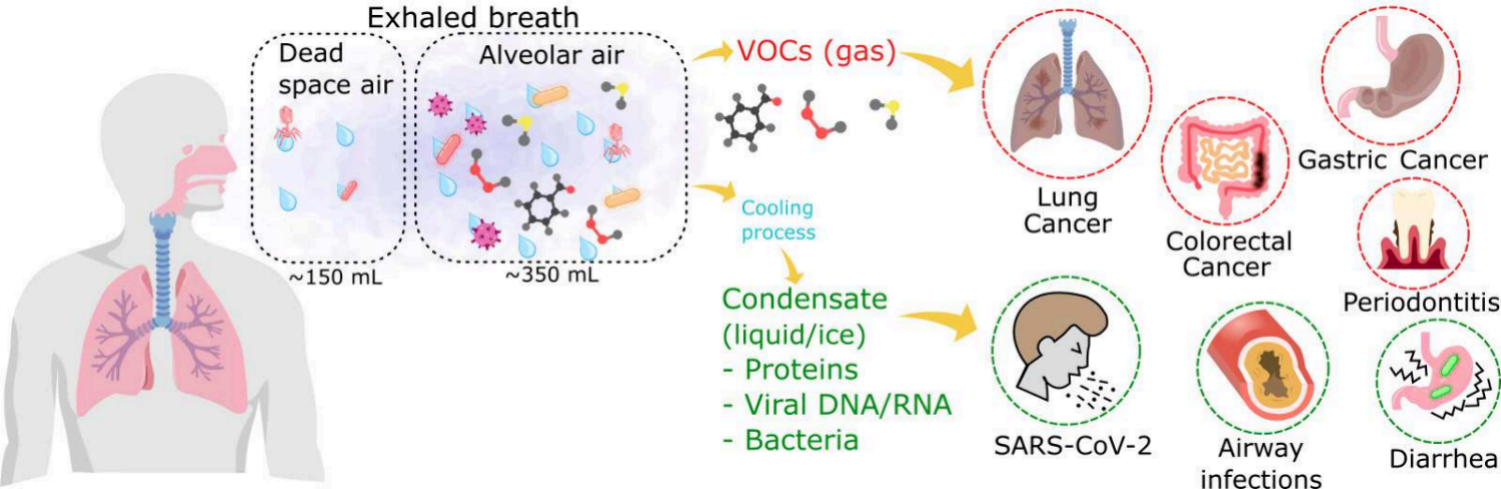
Graphical scheme of the main composition of exhaled breath and the main constituents that can be found in it. In the gaseous phase, VOCs can be found, and these have been used to diagnose different types of cancer. Moreover, breath aerosols can be used to obtain condensates that contain proteins, viruses, and bacteria whose monitoring and detection have led to the diagnosis of, e.g., SARS-CoV-2, airway infections, and diarrhea.

Several chronic illnesses (such as different types of cancer, diabetes, or cardiac conditions) are life-threatening and require daily monitoring. Typically, they are diagnosed using time-consuming and relatively expensive analytical techniques that require trained personnel, and some of them involve invasive and painstaking sample collection procedures. In contrast, one of the main advantages of BA is the essentially limitless amount of sample that can be obtained in a noninvasive and rapid collection. Nevertheless, a bottleneck arises once the samples are obtained, which is the very low concentrations of the biomarkers contained in exhaled breath. In fact, the average concentration of the biomarkers contained in exhaled breath ranges from parts per billion (ppt) to parts per million (ppm).^5^ Table S1 in the Supporting Information shows some of the most abundant VOCs present in human exhaled breath and their corresponding average concentrations; for a more detailed list of the VOCs present in human breath, see ref 6. As can be seen, the concentrations are extremely low; hence, extremely sensitive techniques are required to detect these analytes in such low concentrations.

BA benefits from several well-established and highly sensitive analytical techniques, with Gas Chromatography–Mass Spectrometry (GC-MS) widely regarded as the gold standard.^7^ GC-MS operates by first separating breath into its individual components using gas chromatography followed by mass spectrometry, which identifies each component based on its mass-to-charge ratio. Other MS-based techniques include Proton Transfer Reaction-Mass Spectrometry (PTR-MS) and Selected-Ion Flow Tube-Mass Spectrometry (SIFT-MS).^8–10^ PTR-MS ionizes VOCs in breath samples by using protonated water ions through a proton-transfer reaction, while SIFT-MS uses carefully selected reagent ions to react with VOCs in a controlled manner. After these processes, a mass spectrometer is used to perform the detection. Additionally, Ion Mobility Spectrometry (IMS) is commonly employed in BA.^11^ IMS separates and identifies ions based on their mobility through a gas under an electric field, allowing it to distinguish compounds with similar masses but differing structures.

Despite their high sensitivity, these techniques have limitations. Mass spectrometry-based methods rely on costly equipment, complex sample preparation, and trained operators, which can hinder their practicality for widespread use. As alternatives, emerging methods such as electronic noses (E-noses) and electrochemical sensors offer high sensitivity to detect VOCs in the parts per billion (ppb) range and enable real-time analysis.^12,13^ However, these approaches also face challenges, including difficulties in recognizing VOC patterns and achieving sufficient selectivity in complex mixtures found in breath.

With these considerations in mind, SERS has emerged as a highly promising technique for BA. SERS offers exceptional sensitivity and selectivity, detecting trace compounds in breath at levels comparable to those of MS-based methods. Unlike these traditional approaches, SERS stands out with its straightforward sample preparation, cost-effectiveness, and ability to perform real-time analysis, making it an ideal candidate for advancing breath analysis applications. Because of this, SERS has become an outstanding analytical platform with applications in different fields such as chemistry, catalysis, biology, food science, diagnosis, and biomedicine.^14–18^ By taking advantage of the “hot spots” near the surface of metallic plasmonic nanostructures, a localized surface plasmonic resonance effect is produced. The interaction of the analyte with the hot spots generates an electromagnetic enhancement that produces a tremendous amplification of the corresponding Raman signals by several orders of magnitude (e.g., 10^5^–10^10^), thereby endowing the capability to detect analytes at the single molecule level.^19^ Hence, SERS is considered a powerful analytical technique with high specificity and sensitivity in detecting biomarkers since the resultant SERS spectrum is a fingerprint featured by the analyzed chemical species. Some conventional SERS substrates involve colloidal metallic nanoparticles (NPs), metal NPs dried on solid substrates, and 2D metallic nanoarrays.^20,21^ However, cutting-edge SERS substrates include 3D architectures made of paper or polymers, which are also soft or flexible, thereby facilitating simple sample collection.^22^ Moreover, there have also been breakthroughs in developing increasingly sensitive SERS substrates, with a limit of detection up to the yoctomolar range.^23–25^ Importantly, another advantage of SERS measurements is that they can be readily carried out in real-time and online, thereby offering an excellent (bio)­analytical platform for point-of-care and personalized heathcare applications. For example, BA via SERS has been employed to diagnose different types of cancer, SARS-CoV-2, cystic fibrosis, diarrhea, and periodontitis, among other conditions, see Figure 1.

The existing literature highlights the main techniques for BA and discusses different pathologies that can be diagnosed (such as lung cancer, diabetes, and renal disease) and their corresponding biomarkers.^1,4,26–28^ In addition, the literature also underscores different applications of SERS in human health,^29–33^ or gas molecules in environment and healthcare.^34^ Our Tutorial offers essential and in-depth information to understand the entire process of BA using SERS. In particular, we cover key features of common breath sampling methods and highlight their advantages and disadvantages. We also discuss different types of SERS substrates for BA and their relevant characteristics for specific healthcare applications. Additionally, we explore the employment of machine learning approaches for data postprocessing, outlining their benefits in providing smarter and more efficient diagnostics. Finally, we critically discuss the opportunities, challenges, and future directions of BA using SERS.

## Breath Characteristics

On average, the volume of exhaled breath is approximately 500 mL and mainly consists of gases and aerosols combined and can be further divided into two mixtures: “dead space air” and “alveolar air”.^27^ Dead space air, which constitutes the first 150 mL of exhaled breath, comes from the airways and gastrointestinal tract, where no blood–air exchange takes place.^35^ This portion is sampled when substances from these areas are of interest. It is typically collected through mixed expiratory or total breath sampling, where the complete volume of exhaled breath is analyzed.^36^ In contrast, alveolar air makes up the remaining 350 mL at the end of the breath. This air originates from the lungs and is in contact with the bloodstream. This fraction is clinically relevant because, during the breathing process, a chemical equilibrium is established between the alveolar air and the pulmonary capillary blood.^37^ This equilibrium allows for the exchange of several molecules. Consequently, alveolar air contains diverse VOCs (Table S1). Some VOCs are common and maintain relatively consistent concentrations in exhaled human breath, while others are closely associated with metabolic processes. For instance, abnormal levels of ammonia may indicate kidney failure or liver dysfunction,^38^ and an increase in acetone concentration can be linked to type II diabetes.^39^

Exhaled breath is also warmed and humidified as it travels through the airways. This generates aerosols that can be further condensed as they leave the body by a cooling process. Depending on the application, the exhaled breath condensate can be obtained as liquid or ice.^40^ This condensate primarily consists of water vapor, nonetheless, nonvolatile molecules, proteins, viruses, and bacteria have also been found.^41–43^ Exhaled breath condensate is collected during quiet breathing, where patients typically wear a face-mask or breathe into a collection device for a specific time (minutes in most cases), and after that, the condensate is further analyzed.^44^

As discussed above, exhaled breath is a complex matrix that primarily includes nitrogen, oxygen, carbon dioxide, water, inert gases, and thousands of compounds, including VOCs, exogenous and endogenous compounds, proteins, bacteria, and viruses.^1,45^ Among all the constituents of the breath, it is essential to note that while some components, such as acetone^46^ and isoprene,^9^ have concentrations in the parts per million range, the vast majority of VOCs exist in the parts per billion and parts per trillion range.^5^ SERS substrates can overcome this problem given their single-molecule sensing capabilities.^25^ However, BA using SERS faces several challenges due to the intrinsic characteristics of the human exhaled breath, for instance, the compounds contained in the breath can differ due to various external factors, including the concentration of contaminants in ambient air, dietary habits, smoking, and drinking which complicates the sensing of specific biomarkers.^4^ In addition, VOCs usually have a low affinity and adsorbance in SERS substrates due to the high mobility of gaseous molecules and their small cross-section (which is a measure of the probability of a molecule undergoing a Raman scattering event by relating the intensity power produced by the Raman event with the incident power density provided by the employed laser).^47^ This generates most of the reported SERS substrates to be functionalized with a specific molecule that reacts with the target biomarker, for instance, 4-aminothiophenol which reacts with aldehydes, a common cancer biomarker.^48^ Other research teams have reported biomarkers associated with diseases diagnosed with BA. For example, octanal, cyclopentane, acetone, and methyl ethyl ketone have been identified as biomarkers for various types of cancer, sulfides for halitosis, and nitrogen oxide for asthma. A comprehensive list of diseases and their corresponding BA biomarkers can be found in the literature.^1^

## Breath Sampling

Collecting breath samples is a vital step in employing BA as a diagnosis tool. In the context of SERS applications, it is important to ensure constant and proper exposure of the SERS substrate to the breath in order to maximize the adsorption of the analytes. The corresponding exposure time depends on several factors such as the analyte and the sampling method; for this reason, there is no standard sampling method in BA using SERS. Nonetheless, different examples of sampling methods will be discussed below. Three setups are mainly employed for collecting breath samples in SERS applications, involving the usage of chamber or storage devices, breathalyzer-like devices, or wearable SERS substrates, see Figure 2.

**Figure 2:**
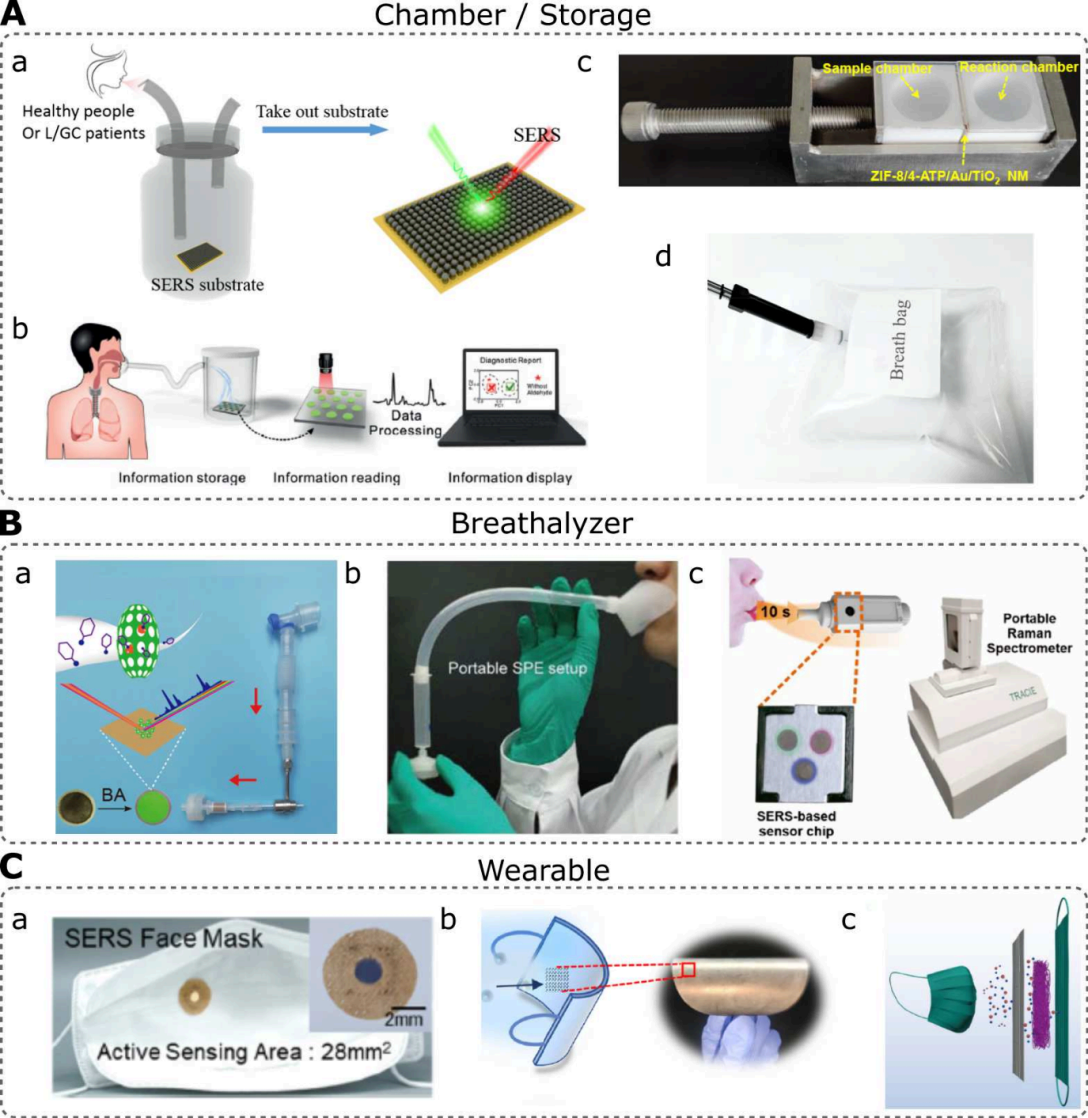
Main setups for BA using SERS. (A) Breath recollection in (a, b, and c) reaction chambers and (d) storage devices such as Tedlar bags. (a) Reprinted in part with permission from ref 49. Copyright 2024 Elsevier. (b) Reprinted in part with permission from ref 51. Copyright 2023 American Chemical Society. (c) Reprinted in part with permission from ref 50. Copyright 2019 John Wiley and Sons. (d) Reprinted in part with permission from ref 53. Copyright 2022 American Chemical Society. (B) (a) Breathalyzer-type devices based on a paper-based thin-film microextraction. Reprinted in part with permission from ref 55. Copyright 2022 American Chemical Society. (b) A portable solid phase extraction device. (c) A chamber to improve breath collection. Reprinted in part with permission from ref 57. Copyright 2022 American Chemical Society. (C) SERS wearable sensors placed in face-masks. (a) Reprinted in part with permission from ref 58. Copyright 2022 American Chemical Society. (b) Reprinted with permission from ref 59. Copyright 2024 American Chemical Society. (c) Adapted with permission from ref 60. Copyright 2024 Elsevier.

### Chambers and Storage Devices

The most common sampling method involves the use of breath-storage devices. For this approach, in the case of real samples, the breath is collected by having the patient exhale into a chamber or breath bag. In the case of prepared-in-lab samples, the chamber is filled with a specific target molecule or a mixture of VOCs. In both cases, the aim is to impregnate the SERS substrate with VOCs, and the analytes should be located within the hotspots of the SERS substrate. For instance, Xie et al.^49^ designed an exhaled breath sample collection device, see Figure 2A-a. The patients were required to blow into the chamber through a disposable mouthpiece for 5 s to eliminate the air in the chamber; the patients breathed then slowly for 30 s so the chamber was filled with breath (about 300 mL). Lastly, SERS measurements were carried out 2 days after breath collection. Likewise, Xuezhi Qiao et al.^50^ utilized a homemade reaction chamber to capture aldehydes with a functionalized SERS substrate, see Figure 2A-b. Another chamber-based method employs an H-type box with a sample and reaction chamber, see Figure 2A-c.^51^ This device was used to impregnate a SERS substrate with gaseous aldehydes. The SERS substrate was placed between the two boxes, and an aldehyde gas environment was produced using a hot plate. The exposure time was 5 min; after this, the SERS substrate was removed for SERS measurements. Another common method to store breath is using Tedlar sampling bags, see Figure 2A-d. The advantage of these bags is that the breath can be stored for days, and the SERS measurements can be then performed. The same as in the aforementioned example of chamber devices, the dead-space air was discarded and the patients were required to blow until the Tedlar bag was full.^52–54^

### Breathalyzers

Breathalyzer-type devices take advantage of the pressure and velocity at which the breath is exhaled. The patient blows into a pipe, and the gases travel through one or different conducts until reaching and passing directly throughout the SERS substrate to capture the target molecule or VOCs. After this, SERS measurements can be performed. For example, Zhaoping Xia et al.^55^ developed a vapor-generated paper-based thin-film microextraction device for the concentration and selective detection of volatile benzaldehyde in human exhalation. Besides performing SERS measurements, they also developed a fluorescence sensor for a dual-modal sensing platform. The breathalyzer-type device is shown in Figure 2B-a. The device is L-shaped. On the top, there is a mouthpiece connected through a flexible tube, a T-shape connector, and a T-shape connector to a filter paper holder where the SERS substrate is placed. This design facilitates air flow through the substrate, enhancing the absorbance of the analytes within the substrate. Following this path of aldehyde detection, a portable solid phase extraction device was designed, see Figure 2B-b.^56^ This device consisted of a disposable mouthpiece, which is connected by a silicon tube to a filter where the SERS substrate is placed. The patients were required to breathe into the mouthpiece for about 2 min in order to concentrate the analytes in the solid phase extraction device. After this, the SERS substrate was removed, and a portable Raman spectrometer was used for SERS detection. In addition, Shi Xuan et al.^57^ developed a breathalyzer that contained a SERS-based sensor chip embedded within a custom-made, hand-held, single-use breath chamber to facilitate the collection of breath samples. This device was designed for SARS-CoV-2 detection. The participants were required to blow continuously into the breath chamber for 10 s, and then SERS measurements were obtained using a portable Raman spectrometer. This type of sampling method allows the rapid recollection of breath as well as the usage of portable Raman spectrometers for point-of-care diagnostic applications.

### Wearable SERS Substrates

Wearable SERS substrates are commonly placed in a face-mask due to the convenience of the constant exposure to exhaled breath gases and aerosols. Wearable SERS face-masks were first applied for the analysis of breath aerosols, which contain several types of proteins and pathogens that can be crucial for early disease diagnosis. Hwang et al.^58^ reported a direct and label-free detection of made-in-lab SARS-CoV-2 spike protein aerosol samples using a highly sensitive SERS substrate on a face-mask. To test their approach, they utilized nebulized samples to emulate the breath aerosols and an exposure time of 10 s, which is equivalent to a 4 h preconcentration of aerosols from respiration. Figure 2C-a shows the implementation of the SERS substrate on the face-mask. Shi et al.^59^ designed a portable platform for rapid pathogen capture of aerosols and direct SERS identification. The platform was tested with a solution of biological samples of *Escherichia coli* and *Pseudomonas aeruginosa* with a concentration equivalent to a 2 h preconcentration of breathing. They successfully classified these two bacteria with high sensitivity. Figure 2C-b displays the face-mask and the SERS substrate reported in this work. It is worth emphasizing that the last two works are label-free SERS sensors tested with prepare-in-lab samples, which do not consider the breath complexity that can interfere with the detection. In addition, the same sampling approach has also been applied to detect gas biomarkers on real samples. For example, Zhang et al.^60^ designed a highly absorbent, sensitive, and functionalized SERS substrate to monitor the levels of hydrogen sulfide in exhaled breath. The SERS substrate was cut into a 3.5 cm × 3.5 cm square and assembled over the face-mask. Figure 2C-c shows a schematic representation of the assembly process and how the substrate is exposed to the breath. They recollected real breath samples from a series of volunteers with different health conditions and under different temperature and humidity ranges. They found that the minimum exposure time for repeatable SERS measurements was 20 min after wearing the mask. The SERS substrate had a high tolerance to environmental conditions, and they were able to classify between healthy and unhealthy volunteers. Wearable SERS substrates present certain advantages, such as an easy collection of the sample and constant monitoring given the continuous exposure to the breath with possibility of real-time analysis;^61^ nevertheless, these methods require longer exposure times compared with the aforementioned methods, and for label-free detection, several factors (such as ambient air contaminants) can interfere with the analysis.

## SERS Substrates in BA

The performance of SERS substrates in biomedical applications strongly depends on their plasmonic properties, surface chemistry, and architecture; thus, the design and fabrication of property-specific substrates are essential for particular biomedical applications. In general, SERS substrates can be made by using nanostructures onto solid substrates or 2D metallic nanoarrays. Recent approaches also involve architectures made of paper- or polymer-based materials that offer a porous and flexible substrate. In the context of BA, nanostructured noble-metal (Au and Ag) substrates are the standard option for SERS substrates; however, these must overcome various challenges, including low analyte concentration, interference from external factors (e.g., oral, smoking, or diet status as well as environmental factors such as temperature, humidity, or air pollution),^2,62^ low affinity, and small analyte cross-section. Furthermore, depending on the application, the substrate must be able to capture and concentrate the sample efficiently. Consequently, SERS substrates typically exhibit several critical features that enhance their reliability and effectiveness in BA for diagnostic purposes. These include porosity, which improves analyte capture and boosts sensitivity, and multifunctionality, which enhances selectivity by detecting target analytes while addressing the significant interference posed by the complex mixture of compounds in breath. Similarly, attributes such as flexibility and reusability have recently gained attention for their potential to develop point-of-care, cost-effective, and sustainable BA diagnostic platforms. Therefore, different types of substrates have been explored for disease screening using BA based on SERS. This section summarizes inspiring examples of SERS substrates employed in BA, focusing on key features and their specific characteristics, such as sensitivity, specificity, and functionalization, for reliable biomedical BA applications.

### Functionalized SERS Substrates

Literature highlights two approaches to detect an analyte using SERS, direct detection, and indirect detection. Direct detection, also known as label-free detection, involves identifying specific Raman bands in the spectrum, including those coming from interfering agents. The indirect approach involves tracking changes in the well-defined Raman bands of a molecule that selectively reacts with the analyte.^63^ This molecule is termed a functionalization molecule or label. A standard method to functionalize a SERS substrate is by immersing the substrate in a solution containing the functionalizing molecule, followed by removing the excess through centrifugation and deionized water or ethanol washes.^51,56^ Figure 3A shows a representative scheme of both direct and indirect detection and also the typical Raman spectrum obtained in each case.

**Figure 3:**
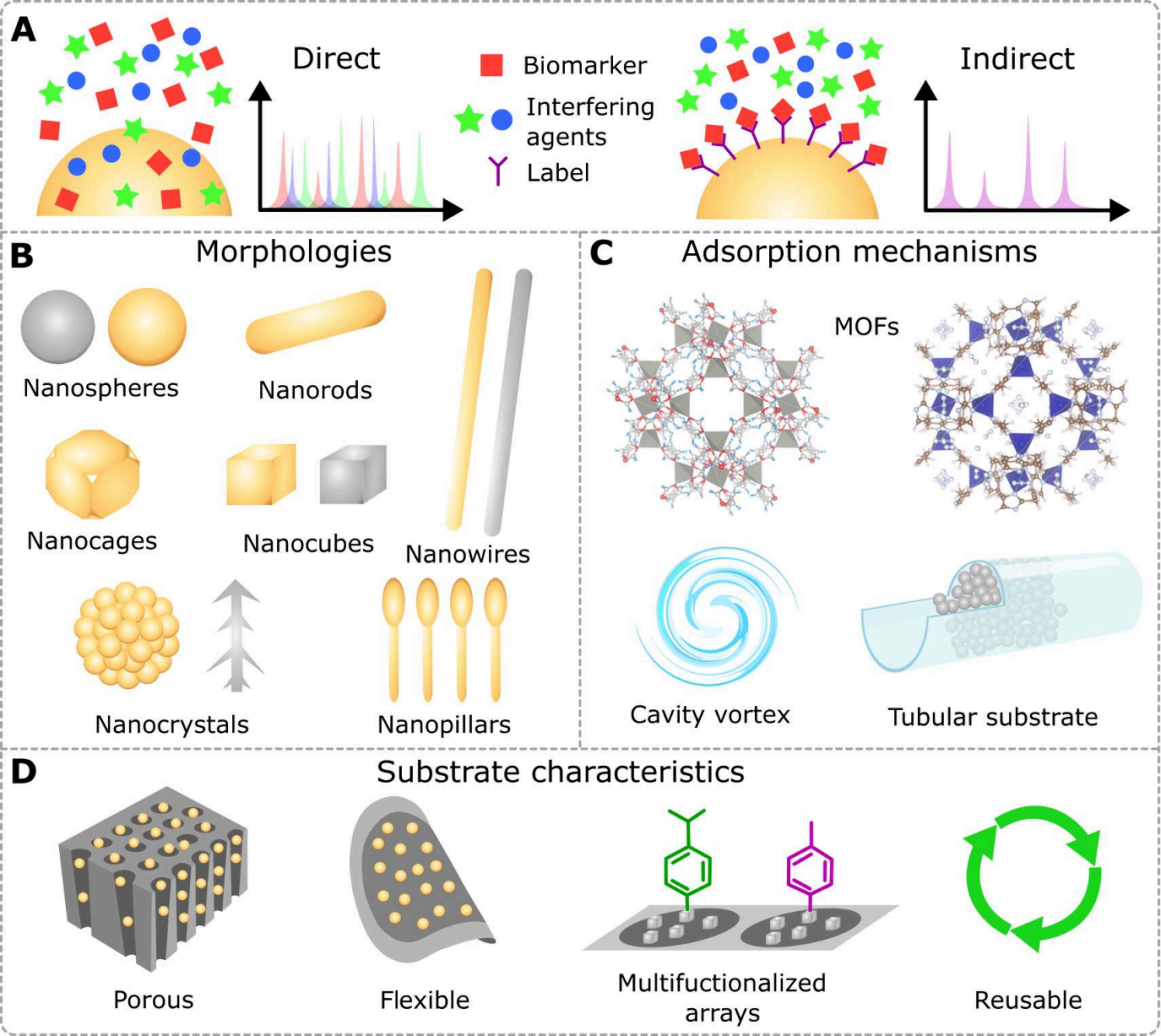
(A) General scheme of the working principles of direct (label-free) and indirect (labeled) SERS analysis. (B) Nanostructure morphologies employed in SERS substrates for BA. (C) Adsorption mechanisms that improve sensitivity, where nanostructures can be combined with either MOFs, cavities inducing vortex effects (due to turbulent airflow), or tubular substrates. (D) Architectures of SERS substrates employed in BA.

Taking into account the discussion above, the main challenge in BA is the accurate detection of specific VOCs given that breath is a complex mixture and the biomarkers are present in very low concentrations. Therefore, a typical approach to tackle this is the functionalization of plasmonic nanostructures in order to produce specific reactions between the functionalization molecules and the biomarkers.

The most commonly reported functionalization molecule in BA based on SERS is 4-aminothiophenol (4-ATP, H_2_NC_6_H_4_SH), which is widely used to detect aldehydes selectively, particularly for cancer screening. In this particular case, the detection of the C=O bond in aldehydes is challenging due to its small Raman cross-section. However, by functionalizing the SERS substrate, a condensation reaction occurs between the −NH_2_ and −CHO groups, forming a C=N bond. The stretching vibration of this bond generates a new peak at 1621 cm^–1^ in the Raman spectra, facilitating the qualitative and quantitative detection of aldehydes.^64,65^ The functionalization method allows for the detection of different types of disease-specific biomarkers for both real and simulated breath samples due to the high specificity provided by the specific functionalization molecules. To this end, different nanostructure morphologies, including nanoparticles,^49,52,56^ nanowires,^48,50,59,66^ and nanocubes^57,61,67,68^ and more complex morphologies such as nanopillars,^69^ nanopyramids,^54^ and dendritic-like nanocrystals,^70^ have been employed and functionalized with 4-ATP, see Figure 3B. The reported morphologies are mainly designed to enhance the SERS performance of the substrate (that is, to improve the enhancement factor and the limit of detection). Some of them were also designed to maximize analyte–substrate or light–substrate interactions, eventually to improve the possibility of capturing the analyte within the hot spots and/or boost the light–molecule interaction.^54,71^

Functionalization imparts high specificity to the SERS substrate; however, to further enhance analytical performance, various strategies have been employed to improve substrate sensitivity by increasing their adsorption capacity. These include coating or decorating the nanostructures with materials such as graphene oxide^52^ or metal–organic frameworks (MOFs),^50,72^ for instance, zeolitic imidazolate framework (ZIF), ZIF-67, and NU-901.^48,51,53,55,66,73^ These porous materials not only offer high surface area but also induce cavity–vortex effects, generating turbulent airflow at the breath–substrate interface and enhancing the analyte interaction. Some examples of this implementation are the work of Qiao et al.^50^ who fabricated a core–shell 3D SERS structure of gold particles coated with a ZIF shell for selective and quantitative detection of gaseous aldehydes, and the work of Zhang et al.^70^ who employed dendritic Ag nanocrystals as a cavity–vortex generators to enhance analyte–substrate interactions. Another effective strategy involves using permeable substrates, such as porous or tubular substrates, that serve as a gas flow channel and a detection chamber,^53^ allowing breath to pass through, facilitating greater interaction with the active surface. For example, a 2D double-opened tube structure of TiO_2_ nanochannel membrane covered with ZIF-8 MOF and Au nanoparticles on the surface has been reported.^51^ This design slows the diffusion of target molecules through the nanochannels, thereby enhancing their retention and local concentration at the sensing surface. As a summary, these approaches and strategies primarily aim to increase the surface area of the substrate and the exposure time, thereby enhancing the probability of capturing the target biomarkers, see Figure 3C.

Building on this, substrate arrays have also been developed to expand the range of detectable biomarkers. By incorporation of multiple independently functionalized substrates within a single platform, these arrays enable the simultaneous detection of various analytes, thereby enhancing diagnostic accuracy and offering a more comprehensive assessment of the breath profile. This approach was used to engineer a microfluidic chip with three detection units that worked by either physisorption or chemisorption.^67^ The first unit (Au@Ag@Au nanocubes) responded to aromatic compounds through a label-free SERS measurement. The second unit (Au@Ag@Au nanocubes) was functionalized with 2,4-dinitrophenylhydrazine to capture aldehydes or ketones. Lastly, the third unit (Au@Ag nanocubes) detected hydrogen sulfide by forming Ag–S covalent bonds. In a similar manner, a point-of-care SERS breathalyzer for the rapid screening of COVID-19 was developed. The SERS substrate consisted of three differently functionalized Ag nanocube substrates. The functionalizing molecules were 4-mercapto­pyridine, 4-mercapto­benzoate, and 4-aminothiophenol to interact with alcohols, ketones, and aldehydes, respectively. This multifunctionalization enhanced the detection by promoting chemical interactions of the biomarkers with the functionalization molecules through hydrogen bonding, ion–dipole interactions, and π–π interactions. These chemical interactions result in a change in the Raman signature.^67^ In this manner, the multifunctionalization allows for the simultaneous detection of various biomarkers and a more precise disease screening.^71^ Other types of functionalization molecules have been explored. Some examples are 4-mercapto­aniline and 2,4-dinitrophenyl­hydrazine which have been used for aldehydes, ketones, or acetic acid detection,^67,68^ 4-mercatopyridine for alcohols detection,^57^ 4-mercaptobenzoate for ketones detection,^57^ and 4-nitrothiophenol (4-NTP) to detect hydrogen sulfide.^60^

Although the main efforts in designing functionalized SERS substrates for BA have been focused on sensitivity and selectivity capabilities, endowing the substrate with characteristics such as porosity, flexibility, and multifunctionalization, other approaches are focused on the renewability of the SERS substrate, see Figure 3D. Renewable porous hierarchical CuFeSe_2_/Au heterostructure nanospheres were prepared by photoreduction. The authors discussed that given the photocatalytic activity, the substrate could provide efficient renewable properties by photodegrading undesired adsorbed biomolecules.^74^ Similarly, another work proposed a multifunctional Ag NPs@ZIF-67/g-C_3_N_4_ solid phase extraction (SPE) membrane,^56^ where the authors highlight that the self-cleaning ability of the substrate (controlled by the photocatalytic characteristics of C_3_N_4_) allows for the reuse of the membrane for future SERS measurements. This characteristic enables the SERS substrate to be recycled, offering sustainability and an ecofriendly character by reducing disposable waste for each measurement.

### Nonfunctionalized SERS Substrates

Direct SERS analysis involves collecting Raman signals of all chemical species adsorbed onto the substrate surface, see Figure 3A. While most SERS substrates in BA are functionalized, some approaches are focused on a label-free approach. A recent approach employed real and simulated breath samples to identify early and advanced gastric cancer. The authors fabricated a AuNPs-decorated reduced graphene oxide SERS substrate supported on an Au film and glass.^52^ Using GC-MS, exhaled breath samples from healthy and gastric-cancer-confirmed patients were analyzed. The authors identified 14 VOCs as biomarkers for early and advanced gastric cancer. The SERS sensor was used as a proof-of-concept, with the simulated and real breath samples of the VOCs identified. The Raman spectra provided by the sensor revealed well-defined differences between healthy and cancer-confirmed patients. Through principal component analysis, the authors were able to classify healthy, early-stage, and advanced-stage gastric cancer patients. In another cancer screening approach, a substrate of Ag nanowires coated with ZIF-8 core–shell nanochains was developed. This substrate contained numerous cavities that slowed down the airflow and increased the exposure time of the sample.^66^

Label-free SERS detection has also been used for breath aerosol analysis and bacteria screening. Using an Au-TiO_2_ nanocomposite substrate, SARS-CoV-2 spike proteins were detected in simulated breath samples.^58^ The authors discussed that the nanocomposite of Au and TiO_2_ generated a high surface energy, which improves adsorption. This configuration enabled the detection of SARS-CoV-2 spike proteins in artificial respiratory aerosols at a 100 pM concentration level. The substrate was placed on a face-mask, which facilitated the constant preconcentration of the aerosols. This approach has also been employed for diarrhea detection using Ag nanowires on a filter membrane.^59^

There are noticeably fewer studies on label-free SERS detection compared with those that utilize functionalized SERS substrates. When comparing these two approaches, both direct and indirect detection have their own advantages and disadvantages in BA. Functionalized substrates offer high sensitivity and specificity, making the quantitative analysis process simpler; moreover, the functionalization allows for a considerable reduction of the background interference produced by the complex mixture represented by breath. This is achieved by keeping track of specific peaks in the spectrum related to particular reactions related to the biomarker. However, they require prior knowledge of the biomarkers or labels, and their fabrication can be quite complex. In contrast, label-free substrates enable direct analysis of a broad range of biomarkers in a more straightforward manner. The main drawbacks of this approach are that the Raman signals of the biomarkers can be overlapped with those of endogenous or exogenous interfering agents and signal-to-noise ratios can be low. This issue is evident, as most existing research on label-free methods has concentrated on testing these substrates with simulated or lab-prepared samples. However, as discussed below, AI algorithms can be applied to deal with such complex Raman spectra. Ultimately, the choice of the detection approach is heavily dependent on the specific application. An extended summary of the different types of SERS substrates employed in BA, including critical features such as functionalization molecule, detected biomarker associated with the targeted disease, limit of detection, and acquisition parameters, are highlighted in Table S2 in the Supporting Information.

## Healthcare Applications

The composition of VOCs in human exhaled breath can vary significantly from person to person due to several factors, including lifestyle, physical condition, and environmental influences. However, certain VOCs are commonly found in individuals with specific health conditions or diseases. This means that even subtle changes in either the concentration or composition of breath can be detected and linked to particular diseases.^75^ This section summarizes the main types of diseases that have been detected or screened using BA and SERS substrates.

### Cancer

Cancer is one of the diseases with the highest mortality rates worldwide, making early detection critically important in the medical field.^76^ Common methods for diagnosing cancer include imaging examinations, tissue biopsies, and blood tests. However, these tests can often be invasive and uncomfortable for patients. Recently, BA for cancer diagnosis has emerged as a promising tool, having identified and correlated over 100 VOCs with various types of cancer.^77^ Most of the studies involving BA via SERS primarily focus on cancer types related to the respiratory and gastrointestinal systems. This emphasis is largely due to the convenience of detecting biomarkers through exhaled breath. Moreover, among the various biomarkers reported for cancer, an increase in aldehyde concentration is frequently mentioned. Cancer cells typically exhibit high metabolic activity and rapid growth, leading to increased oxidative stress. This oxidative stress results in lipid peroxidation within cell membranes, producing aldehydes as byproducts.^78^ By detecting aldehydes, different types of cancer have been successfully screened, for instance, lung cancer,^49–51,56,64,70,73,74,79^ gastric cancer,^49,53,80,81^ and colorectal cancer.^48^ However, studies utilizing different functionalization molecules, such as 2,4-dinitrophenylhydrazine (DHPH) and 4-mercapto­aniline (PATP), or even employing label-free detection methods, have reported positive results in cancer screening, including gastric^52^ and oral cancer.^66^

### COVID-19

The outbreak of the COVID-19 pandemic in 2019 highlighted the urgent need for clinical devices capable of rapid and large-scale disease screening.^82^ Among the emerging methods, BA using SERS has been explored as a potential approach for COVID-19 screening.^83^ In 2022, Leong et al.^57^ reported the first point-of-care breathalyzer-type devices for COVID-19 screening. The authors discussed how immune responses and metabolic changes induced by coronavirus modify the concentrations of aldehydes, ketones, and alcohols. Based on this observation, they proposed a multifunctional SERS array substrate using Ag nanocubes. They selected 4-mercaptobenzoate to study its interaction with alcohols in the spectral range of 490–550 cm^–1^, 4-mercatopyridine to interact with ketones in the range of 1560–1680 cm^–1^, and 4-aminothiophenol for aldehyde detection in the region of 1050–1500 cm^–1^. This setup allowed for the construction of a superprofile, whereby monitoring specific peak changes enabled accurate diagnoses within 5 min. In a different study, Hwang et al.^58^ developed a face-mask SERS substrate for the detection of SARS-CoV-2 in artificial breath aerosols. This work was motivated by the fact that COVID-19 is primarily transmitted through respiratory droplets. The authors focused on detecting SARS-CoV-2 spike proteins using a label-free approach accompanied by machine learning techniques, resulting in a rapid, robust, and facile screening method for the early diagnosis of COVID-19.

### Other Types of Diseases

The screening of airway infections in cystic fibrosis patients was a pioneer development of BA via SERS. Cystic fibrosis leads to the production of thick, sticky mucus that primarily accumulates in the lungs.^84^ This condition provides an environment for the growth of various bacteria, including *P. aeruginosa*. This particular bacterium is the most common cause of chronic airway infections and can even lead to the death of the patient. *P. aeruginosa* releases hydrogen cyanide, a toxic gas, as a competitive mechanism with other organisms, thereby representing a potential biomarker for this bacterium. Lauridsen et al.^69,85^ developed an SERS substrate using gold nanopillars, leveraging the strong affinity of cyanide for metals. They focused on the characteristic Raman peak at 2135 cm^–1^, which corresponds to the C≡N bond. The authors evaluated the performance of their SERS substrate with laboratory-prepared samples and subsequently tested it with real samples, successfully detecting infections at an early stage. They also conducted tests with a mutated strain and provided explanations for why hydrogen cyanide may go undetected in some patients with chronic *P. aeruginosa* airways infections. In a different study, *P. aeruginosa* and *E. coli* were utilized as biomarkers in a label-free approach to detect diarrhea. A face-mask SERS substrate was proposed to preconcentrate the pathogens found in breath aerosols, achieving high specificity and sensitivity in distinguishing between the two bacteria.^59^

Furthermore, another toxic gas that has been used as a biomarker in BA through SERS is hydrogen sulfide, which is linked to periodontitis and halitosis.^86^ In this study, the authors developed a mask-based SERS substrate that was functionalized with 4-NTP. This functionalization allows for a redox reaction with hydrogen sulfide, resulting in a change in the Raman spectrum. The study focused on tracking four specific peaks to monitor this change and achieved a sensitive detection at the ppb level.

As shown in Table S2, the literature is mainly focused on cancer screening using functionalized SERS substrates and aldehydes as biomarkers. Additionally, other targeted diseases primarily include the respiratory and gastrointestinal systems, which are directly related to breath samples. This suggests that for these applications, biomarkers should be generated or closely associated with these systems, as they can be expressed in higher concentrations in breath samples.

## Machine Learning in BA

In recent years, the employment of artificial intelligence (AI) in biomedical science has resulted in significant advancements in diagnostic technologies,^87^ and BA is not the exception.^22,88–90^ Raman spectra in breath samples provide valuable insights into a person’s health status. However, analyzing and extracting meaningful information for diagnosing specific diseases from SERS spectra is challenging due to their complex nature, the spectral overlapping among several breath compounds, and the low signal-to-noise ratios. These challenges can be addressed with AI algorithms. By leveraging the sensitivity provided by SERS and the pattern recognition capabilities of AI, these methods enable precise analysis of breath biomarkers to enhance the accuracy, speed, and reliability of BA, offering an excellent tool for disease screening. This section explores recent applications of AI in BA, and how its implementation has enhanced the diagnostic process.

One of the most utilized machine learning methods in BA is artificial neural networks (ANNs).^81^ For instance, in a recent work, artificial breath samples were prepared to train an ANN to classify healthy people and those with oral cancer.^66^ This model achieved an accuracy of 99%, outperforming other widely used classification methods in BA via SERS such as principal components analysis and partial least-squares discriminant analysis. ANN were utilized by Minghong Li et al.^48^ to identify people with colorectal cancer. Simulated data was also used, and the interaction between aldehydes (biomarker) and 4-ATP (label) results in the emergence of a distinct peak at 1625 cm^–1^, accompanied by additional spectral modifications. These alterations are subtle, making visual spectral analysis challenging. However, the advanced feature extraction capabilities of the ANN enable accurate differentiation between healthy individuals and those diagnosed with colorectal cancer. The model reached an accuracy in the full data set of 93.7%, which yielded better predictions than those offered by either partial least-squares discriminant analysis or principal component analysis with linear discriminant analysis, which were also tested. Literature also reported the detection of lung and gastric cancer using AI-assisted BA via SERS.^49^ The authors collected 1780 SERS spectra, which contained data resulting from real samples of healthy people, lung cancer patients, and gastric cancer patients. The ANN could classify the three health stages with an accuracy of 89%. A more complex ANN model, in particular, an autoencoder was proposed by Charles Hwang et al.^58^ for SARS-CoV-2 analysis. An autoencoder allows feature extraction by reducing the dimensionality of the input into the latent space. This facilitates the clustering and classification of the inputs based on their main characteristics. In this work, the team prepared 5 different concentrations of aerosolized SARS-CoV-2 lysates in an artificial respiratory solution. The data was artificially augmented, and the classification was performed by concentration levels, with an accuracy of 98%.

Other machine learning methods have also been applied to BA using SERS, Jing Xu et al.^51^ assessed six different machine learning algorithms for lung cancer screening. The algorithms were convolutional neural networks, k-nearest neighbors, deep neural networks, random forest classifier, support vector machine, and decision tree. The input data were the SERS spectra of seven types of gaseous aldehydes (typical cancer biomarkers) mixed with eight interfering specimens to simulate real breath samples. The classification accuracy of all of the studied models was above 96%.

Machine learning algorithms, combined with BA via SERS, offer new opportunities for quick and accurate detection of various types of health conditions. Nonetheless, it is important to bear in mind that the detection approach strongly influences the machine learning algorithm to be employed. For instance, unsupervised machine learning algorithms are better suited for label-free detection due to the nature of the data, whereas supervised algorithms can easily be applied to label-based data. Both types of algorithms are capable of effectively processing complex, high-dimensional spectral data. They also assist in extracting relevant features from raw spectral data, which enhances the sensitivity and specificity of BA and allows for the detection of disease-related biomarkers, even at low concentrations. Once trained, these models can be used in real-time for BA, creating an accurate and efficient point-of-care diagnostic platform. Figure 4 summarizes the machine learning approaches that have been used for BA via SERS as well as the diseases that are diagnosed with them.

**Figure 4:**
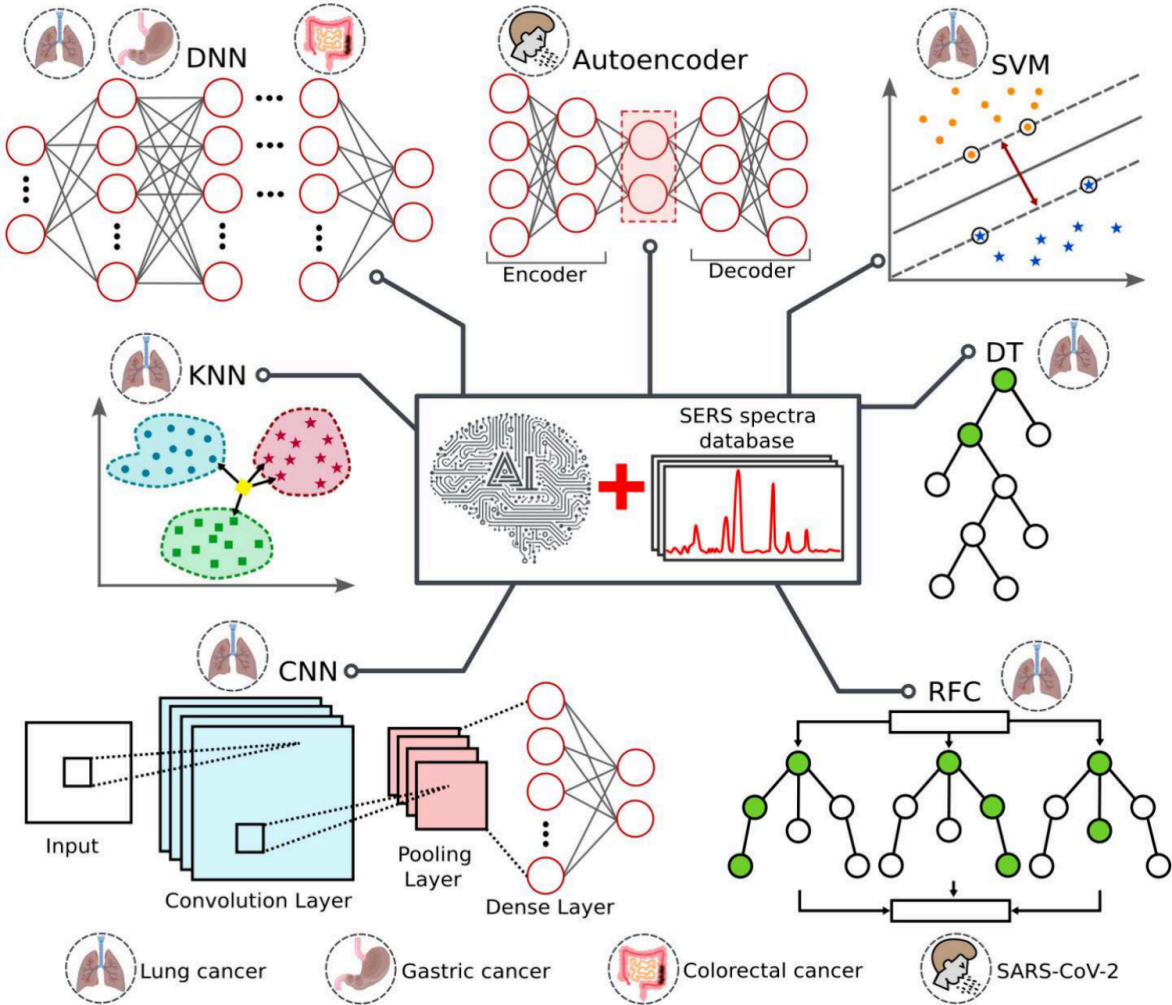
Machine learning algorithms used in BA via SERS. The small icons represent the targeted pathologies. Abbreviations: *DNN*, Deep Neural Networks; *SVM*, Support Vector Machine; *KNN*, k-nearest neighbors; *DT*, Decision Tree; *CNN*, Convolutional Neural Networks; *RFC*, Random Forest Classifier.

## Conclusion and Future Perspectives

We offer a critical overview in the field of BA via SERS, highlighting biomedical applications. BA represents a promising alternative for rapid and noninvasive clinical diagnosis. The employment of SERS as an analytical technique addresses the limitations of conventional methods in breath analysis, which often involve complex and expensive instruments, cumbersome sample preparation processes, and the need for trained personnel. SERS approaches may obviate these limiting factors, as they have been proven to be as sensitive as the conventional techniques. Sampling methods such as chamber storage, breathalyzers, and wearable sensors have been proposed and successfully applied. Besides, a wide variety of nanostructure architectures, functionalizing molecules, and coating materials have been proposed in attempts to improve the sensitivity and accuracy of the sensors for the screening of diverse diseases (mainly different types of cancer and infectious diseases). Postprocessing methodologies such as machine learning algorithms are playing a critical role in dealing with complex data obtained from SERS measurements. Machine learning approaches such as neural networks allow the processing of complex data for feature extraction and classification tasks, facilitating a smart diagnosis procedure. Nonetheless, there are still some challenges that need to be overcome to make BA using SERS a viable and accepted method for clinical diagnostics.

Currently, there is no standardized sampling method for breath analysis using SERS. As previously discussed, breath is a complex mixture that can be easily contaminated by external factors. Therefore, it is essential to establish some standard procedures for the sampling process. For example, most of the discussed approaches implemented sampling methods (acquisition devices, exposure times, and storage) based on their specific requirements. This lack of standardization, along with the wide variety of SERS substrates available, complicates the massive implementation of these methods.

The implementation of label-free approaches for BA is challenging due to the complexity of breath composition. As a result, the literature is mainly focused on functionalized SERS substrates that are generally disease-specific, which limits the range of medical conditions that can be screened. However, the successful implementation of label-free sensing methods combined with machine learning algorithms could allow for a broader range of clinical conditions to be analyzed in a single test. In fact, a promising machine learning approach for complex multiclassification tasks has been recently proposed for bacteria classification.^91^ Due to the complexity of the breath, an adequate adaptation and implementation of this approach could have tremendous potential for breath analysis.

There is currently a gap in the literature regarding the screening of a wide variety of diseases. Most studies focusing on BA using SERS have primarily investigated diseases related to the respiratory and gastrointestinal systems; in particular, lung cancer is the most studied. Further research is needed to determine whether any non-respiratory-related diseases can be screened using BA and SERS, above all considering that a chemical equilibrium is established between the alveolar air and the pulmonary capillary blood. All in all, BA via SERS represents a powerful platform for the next generation of sensors which are expected to provide tools for personalized healthcare and preventive medicine.

## Supplementary Material



## Notes

List of abbreviations used in the paper; table listing common VOCs found in human breath; table summarizing the different types of SERS substrates used in BA (PDF)
