# Supplementary material for: Harnessing Surface-Enhanced Raman Spectroscopy for Breath-Based Diagnostics
Source: Anal Chem. 2025 May 9;97(19):10099–109. doi: 10.1021/acs.analchem.5c00167 (PMC12096349; doi:10.1021/acs.analchem.5c00167)
Supplement: Supplementary file 1 [file ac5c00167_si_001.pdf]

Supporting Information

Harnessing Surface-Enhanced Raman Spectroscopy for Breath-Based Diagnostics

Ivan A. Lujan-Cabrera and Eden Morales-Narváez*

Biophotonic Nanosensors Laboratory, Centro de Física Aplicada y Tecnología Avanzada (CFATA), Universidad Nacional Autónoma de México (UNAM), Querétaro 76230, Mexico

E-mail: eden@fata.unam.mx

Table of Contents

Acronyms S-1
Table S1: Representative VOCs identified in the breath and their average concentrations...S-2
Table S2: Summary of different types of SERS substrates employed in BA.................S-3
Supporting references S-6

Acronyms

4-ATP, 4-aminothiophenol

4-NTP, 4-nitrothiphenol

AI, Artificial Intelligence

ANN, Artificial Neural Networks

BA, Breath Analysis

DHPH, 2,4-dinitrophenylhydrazine

GC-MS, Gas Chromatography-Mass Spectrometry

IMS, Ion Mobility Spectrometry

MOFs, Metal-Organic Frameworks

NPs, Nanoparticles

PATP, 4-mercaptonoaniline

ppb, Parts per Billion

ppm, Parts per Million

ppt, Parts per Trillion

PTR-MS, Proton Transfer Reaction-Mass Spectrometry

SERS, Surface-Enhanced Raman Spectroscopy

SIFT-MS, Selected-Ion Flow Tube-Mass Spectrometry;

SPE, Solid Phase Extraction

VOCs, Volatile Organic Compounds

ZIF, Zeolitic Imidazolate Framework

Table S1: Representative VOCs identified in the breath and their average concentrations.[S1,S2]

Identified VOCs	Concentration
Nitric oxide (NO)	10–50 ppb
Nitrous oxide (N_2O)	1–20 ppb
Ammonia (NH_3)	0-6 ppm
Monóxido de Carbono (CO)	0–6 ppm
Hydrogen sulphide (H_2S)	0–1.3 ppm
Methane (CH_4)	2–10 ppm
Acetone	0.3–1 ppm
Pentane	0–10 ppb
Isoprene	~105 ppb
Aldehydes and ketones	1-1250 ppm

Table S2: Summary of different types of SERS substrates employed in BA.

SERS substrate	Funct molecule	Biomarker	Disease	LOD (ppb)	Sample origin	# of real samples	Exposure time (min)	λ (nm)	Spectral range (Raman shift (cm^{-1}))	Power (mW)	Ref.
Au-coated SiNPis	NA	Hydrogen cyanide	A-In	18	LP	NA	0.5	780	100-3400	5.0	S3
AuNPs on RGO on Au film and glass	NA	Acetone,<br>isopropene<br>tetradecane,<br>2-Methylpentane,<br>3-methylpentane,<br>hexane,<br>2,3-dimethylpentane,<br>phenyl acetate,<br>pivalid acid,<br>hexanel<br>2-methylhexane,<br>3-methylhexane,<br>dodecane,<br>methanol	GC	479	S/R	200	30	785	400-2000	2	S4
Au-coated SiNPis	NA	Hydrogen cyanide	A-In	1.8	R	5	20	1064	100-3000	NR	S5
GSPs@ZIF-8	4-ATP	4-ethylbenzaldehyde	LC	10	S	NA	10	785	800-1850	1	S6
Dendritic AgNCrs	4-ATP	Benzaldehyde	LC	NR	S	NA	5-60	785	800-1800	1	S7
AgNWs@LDH	4-ATP	p-ethylbenzaldehyde	LC	1.9	S	NA	10	785	1000-1700	0.5	S8
CuFeSe2/Au NSs	4-ATP	Benzaldehyde	LC	1.0	S	NA	5-80	785	800-1700	NR	S9
AgNPs@ZIF-67/g-C_3N_4	4-ATP	Benzaldehyde	LC	0.14	S/R	5	50	785	200-2000	10	S10
GNRs-QDs@NU-901	PATP	Benzaldehyde	LC	0.1	S/R	20	5	785	900-1800	10	S11
Au@Ag@Au NCs/ Au@Ag NCs	DNPH, R6G	Acetaldehyde,<br>acetone,<br>benzaldehyde	LC	~ ppb	S/R	23	60	632.8	600-1650	2	S12

AgNPs@ZIF-67	4-ATP	Butanone, n-pentanal, n-hexanal, cyclohexanone, n-octanal, benzaldehyde, n-decanal, glyoxal, methylglyoxal	GC	3	R	118	20	632.8	900-1800	0.1	S13
AgNCs array	MBA MPY 4-ATP	Methanol, ethanal, heptanal, octanal, acetone	COVID-19	~ ppb	S/R	501	2	785	600-1600	50	S14
Au-TiO_2 nanocomposite	NA	SARS-CoV-2 spike proteins	COVID-19	10^1 pfu/mL	S	NA	0.16	633	600-1650	5	S15
Au@$CsPbBr_3$	4-ATP	Formaldehyde, glyoxal, butyraldehyde, glutaraldehyde, caprylic aldehyde, capric aldehyde, 2-furaldehyde, Benzaldehyde, ethylbenzaldehyde	GC	~ ppb	R	30	120	532/785	600-1800	NA	S16
ZIF-8-covered Au/TiO_2NM	4-ATP	4-ethylbenzaldehyde	LC	0.19	S	NA	5	638	800-1800	1.6	S17
AgNW@ZIF-8 core-shell nanochains	NA	Methanethiol	OC	4.81	S	NA	300	632.8	400-3000	0.7	S18
Ag/Si/Ag PMPD chip	4-ATP	4-ethylbenzaldehyde	LC	0.1	S/R	10	30	532	900-1700	2.4	S19
AgNP@ZIF on Au film	4-ATP	Glutaraldehyde	LC, GC	1	S/R	79	0.5	633	1000-1700	0.7	S20

AgNWs@ZIF-8 on PBM	4-ATP	Benzaldehyde	CRC	1.06	S	NA	240	532	400-1800	NR	S21
PVDF/ZnO NWs/Ag/ZIF-8	4-NTP	Hydrogen sulfide	Pt, Ht	0.1	S/R	20	20	523	900-1800	4.8	S22
AgNWs@ on filter	NA	Escherichia coli, Pseudomonas aeruginosa	Diarrhea	10^{-3} cfu/mL	S	NA	NA	785	600-1200	NR	S23
AgNCs	4-ATP	Nitrite	A-In	0.0046	R	1	10	532	200-1800	1.6	S24
AgNCs	4-MBA	Acetic acid	NR	60	S	NA	0-60	785	200-2000	10	S25
T-Si/Al_2O_3/Ag/Au substrate	CTAB<br>4-NTP<br>4-AA	Hydrogen sulfide	Ht, A-In	0.1	R	8	~ 3	532	500-1800	0.48	S26
MesoAu@ZIF-8	4-ATP	Benzaldehyde	LC	0.32	S	NA	20	785	600-1800	3	S27

Acronyms: 4-AA, 4-acetamidobenzenesulfonyl azide; 4-ATP, 4-aminothiophenol; A-In, airway infections; CRC, colorectal cancer; CTAB, Cetyl Trimethyl Ammonium Bromide; DNPH, 2,4-dinitrophenylhydrazine; GC, gastric cancer; GNR, gold nanorod; GSP, gold superparticle; Ht, halitosis; LDH, layer double hydroxide; LG, lung cancer; MBA, 4-mercaptobenzoate; MPY, 4-mercatopyridine; NA, not applicable; NC, nanocube; NCr, nanocrystal; NM, nanomembrane; NP, nanoparticle; NPi, nanopillar; NR, not reported; NW, nanowire; OC, oral cancer; PATP, 4-mercaptonoaniline; PMPD, Porous Micropyramid; Pt, periodontitis; PVDF, polyvinylidene fluoride; R, real; R6G, rhodamine; RGO, reduce graphene oxide; S, simulated.

Supporting references

(S1) Das, S.; Pal, M. Review—Non-Invasive Monitoring of Human Health by Exhaled Breath Analysis: A Comprehensive Review. *Journal of The Electrochemical Society* **2020**, *167*, 037562.

(S2) Kuo, T.-C.; Tan, C.-E.; Wang, S.-Y.; Lin, O. A.; Su, B.-H.; Hsu, M.-T.; Lin, J.; Cheng, Y.-Y.; Chen, C.-S.; Yang, Y.-C.; Chen, K.-H.; Lin, S.-W.; Ho, C.-C.; Kuo, C.-H.; Tseng, Y. J. Human Breathomics Database. *Database* **2020**, *2020*, baz139.

(S3) Lauridsen, R. K.; Rindzevicius, T.; Molin, S.; Johansen, H. K.; Berg, R. W.; Alstrøm, T. S.; Almdal, K.; Larsen, F.; Schmidt, M. S.; Boisen, A. Towards quantitative SERS detection of hydrogen cyanide at ppb level for human breath analysis. *Sensing and Bio-Sensing Research* **2015**, *5*, 84–89.

(S4) Chen, Y.; Zhang, Y.; Pan, F.; Liu, J.; Wang, K.; Zhang, C.; Cheng, S.; Lu, L.; Zhang, W.; Zhang, Z.; Zhi, X.; Zhang, Q.; Alfranca, G.; de la Fuente, J. M.; Chen, D.; Cui, D. Breath Analysis Based on Surface-Enhanced Raman Scattering Sensors Distinguishes Early and Advanced Gastric Cancer Patients from Healthy Persons. *ACS Nano* **2016**, *10*, 8169–8179.

(S5) Lauridsen, R. K.; Sommer, L. M.; Johansen, H. K.; Rindzevicius, T.; Molin, S.; Jelsbak, L.; Engelsen, S. B.; Boisen, A. SERS detection of the biomarker hydrogen cyanide from Pseudomonas aeruginosa cultures isolated from cystic fibrosis patients. *Scientific Reports* **2017**, *7*, 45264.

(S6) Qiao, X.; Su, B.; Liu, C.; Song, Q.; Luo, D.; Mo, G.; Wang, T. Selective Surface Enhanced Raman Scattering for Quantitative Detection of Lung Cancer Biomarkers in Superparticle@MOF Structure. *Advanced Materials* **2018**, *30*, 1702275.

(S7) Zhang, Z.; Yu, W.; Wang, J.; Luo, D.; Qiao, X.; Qin, X.; Wang, T. Ultrasensitive Surface-Enhanced Raman Scattering Sensor of Gaseous Aldehydes as Biomarkers of

Lung Cancer on Dendritic Ag Nanocrystals. *Analytical Chemistry* **2017**, *89*, 1416–1420.

(S8) Qiao, X.; Chen, X.; Huang, C.; Li, A.; Li, X.; Lu, Z.; Wang, T. Detection of Exhaled Volatile Organic Compounds Improved by Hollow Nanocages of Layered Double Hydroxide on Ag Nanowires. *Angewandte Chemie International Edition* **2019**, *58*, 16523–16527.

(S9) Wen, H.; Wang, H.; Hai, J.; He, S.; Chen, F.; Wang, B. Photochemical Synthesis of Porous CuFeSe2/Au Heterostructured Nanospheres as SERS Sensor for Ultrasensitive Detection of Lung Cancer Cells and Their Biomarkers. *ACS Sustainable Chemistry & Engineering* **2019**, *7*, 5200–5208.

(S10) Huang, Y.; Xie, T.; Zou, K.; Gu, Y.; Yang, G.; Zhang, F.; Qu, L.-L.; Yang, S. Ultrasensitive SERS detection of exhaled biomarkers of lung cancer using a multifunctional solid phase extraction membrane. *Nanoscale* **2021**, *13*, 13344–13352.

(S11) Xia, Z.; Li, D.; Deng, W. Identification and Detection of Volatile Aldehydes as Lung Cancer Biomarkers by Vapor Generation Combined with Paper-Based Thin-Film Microextraction. *Analytical Chemistry* **2021**, *93*, 4924–4931.

(S12) Yang, K.; Zhang, C.; Zhu, K.; Qian, Z.; Yang, Z.; Wu, L.; Zong, S.; Cui, Y.; Wang, Z. A Programmable Plasmonic Gas Microsystem for Detecting Arbitrarily Combinated Volatile Organic Compounds (VOCs) with Ultrahigh Resolution. *ACS Nano* **2022**, *16*, 19335–19345.

(S13) Huang, L.; Zhu, Y.; Xu, C.; Cai, Y.; Yi, Y.; Li, K.; Ren, X.; Jiang, D.; Ge, Y.; Liu, X.; Sun, W.; Zhang, Q.; Wang, Y. Noninvasive Diagnosis of Gastric Cancer Based on Breath Analysis with a Tubular Surface-Enhanced Raman Scattering Sensor. *ACS Sensors* **2022**, *7*, 1439–1450.

(S14) Leong, S. X.; Leong, Y. X.; Tan, E. X.; Sim, H. Y. F.; Koh, C. S. L.; Lee, Y. H.; Chong, C.; Ng, L. S.; Chen, J. R. T.; Pang, D. W. C.; Nguyen, L. B. T.; Boong, S. K.; Han, X.; Kao, Y.-C.; Chua, Y. H.; Phan-Quang, G. C.; Phang, I. Y.; Lee, H. K.; Abdad, M. Y.; Tan, N. S.; Ling, X. Y. Noninvasive and Point-of-Care Surface-Enhanced Raman Scattering (SERS)-Based Breathalyzer for Mass Screening of Coronavirus Disease 2019 (COVID-19) under 5 min. *ACS Nano* **2022**, *16*, 2629–2639.

(S15) Hwang, C. S. H.; Lee, S.; Lee, S.; Kim, H.; Kang, T.; Lee, D.; Jeong, K.-H. Highly Adsorptive Au-TiO2 Nanocomposites for the SERS Face Mask Allow the Machine-Learning-Based Quantitative Assay of SARS-CoV-2 in Artificial Breath Aerosols. *ACS Applied Materials & Interfaces* **2022**, *14*, 54550–54557.

(S16) Man, T.; Lai, W.; Zhu, C.; Shen, X.; Zhang, W.; Bao, Q.; Chen, J.; Wan, Y.; Pei, H.; Li, L. Perovskite Mediated Vibronic Coupling of Semiconducting SERS for Biosensing. *Advanced Functional Materials* **2022**, *32*, 2201799.

(S17) Xu, J.; Xu, Y.; Li, J.; Zhao, J.; Jian, X.; Xu, J.; Gao, Z.; Song, Y.-Y. Construction of High-Active SERS Cavities in a TiO2 Nanochannels-Based Membrane: A Selective Device for Identifying Volatile Aldehyde Biomarkers. *ACS Sensors* **2023**, *8*, 3487–3497.

(S18) Xie, X.; Yu, W.; Chen, Z.; Wang, L.; Yang, J.; Liu, S.; Li, L.; Li, Y.; Huang, Y. Early-stage oral cancer diagnosis by artificial intelligence-based SERS using Ag NWs@ZIF core–shell nanochains. *Nanoscale* **2023**, *15*, 13466–13472.

(S19) Gao, Y.; Zhu, H.; Wang, X.; Shen, R.; Zhou, X.; Zhao, X.; Li, Z.; Zhang, C.; Lei, F.; Yu, J. Promising Mass-Productive 4-Inch Commercial SERS Sensor with Particle in Micro-Nano Porous Ag/Si/Ag Structure Using in Auxiliary Diagnosis of Early Lung Cancer. *Small* **2023**, *19*, 2207324.

(S20) Xie, X.; Yu, W.; Wang, L.; Yang, J.; Tu, X.; Liu, X.; Liu, S.; Zhou, H.; Chi, R.;

Huang, Y. SERS-based AI diagnosis of lung and gastric cancer via exhaled breath. *Spectrochimica Acta Part A: Molecular and Biomolecular Spectroscopy* **2024**, *314*, 124181.

(S21) Li, M.; He, X.; Wu, C.; Wang, L.; Zhang, X.; Gong, X.; Zeng, X.; Huang, Y. Deep Learning Enabled SERS Identification of Gaseous Molecules on Flexible Plasmonic MOF Nanowire Films. *ACS Sensors* **2024**, *9*, 979–987.

(S22) Zhang, X.; Cai, X.; Yin, N.; Che, Y.; Jiao, Y.; Zhang, C.; Yu, J.; Liu, C. Hierarchical PVDF/ZnO/Ag/ZIF-8 nanofiber membrane used in trace-level Raman detection of H2S. *Journal of Hazardous Materials* **2024**, *471*, 134441.

(S23) Shi, Y.; Fang, J. Directly Self-Assembly of Aligned Ag NWs Films at the Air–Water Interface for the Detection of Pathogens in Artificial Breath Aerosols. *Analytical Chemistry* **2024**, *96*, 2474–2480.

(S24) Chen, C.; Liu, J.; Lu, J.; Wang, Y.; Zhai, J.; Zhao, H.; Lu, N. In Situ Collection and SERS Detection of Nitrite in Exhalations on Facemasks Based on Wettability Differences. *ACS Sensors* **2024**, *9*, 3680–3688.

(S25) Kou, Y.; Zhang, X.-G.; Li, H.; Zhang, K.-L.; Xu, Q.-C.; Zheng, Q.-N.; Tian, J.-H.; Zhang, Y.-J.; Li, J.-F. SERS-Based Hydrogen Bonding Induction Strategy for Gaseous Acetic Acid Capture and Detection. *Analytical Chemistry* **2024**, *96*, 4275–4281.

(S26) Che, Y.; Ni, Y.; Jiao, Y.; Lei, F.; Liu, C.; Zhao, X.; Li, Z.; Zhang, C.; Yu, J. A Strategy for Accurate SERS Gas Detection: Skillful Integration of Mass-Productive Wafer-Scale SERS Substrate and Machine Learning–Assisted Multifeature Profiling. *ACS Photonics* **2024**, *11*, 3331–3342.

(S27) Shi, Y.; Fang, J. Yolk–Shell Hierarchical Pore Au@MOF Nanostructures: Efficient Gas Capture and Enrichment for Advanced Breath Analysis. *Nano Letters* **2024**, *24*, 10139–10147.
